# Effects of Rumen-Protected Lysine and Tannins on Meat Quality and Fatty Acid Profile in Lambs

**DOI:** 10.3390/foods15010049

**Published:** 2025-12-23

**Authors:** Claudiney Felipe Almeida Inô, Roberto Matheus Tavares de Oliveira, José Morais Pereira Filho, Kevily Henrique de Oliveira Soares de Lucena, Lucas de Souza Barros, Ronaldo Lopes Oliveira, Claudio Vaz Di Mambro Ribeiro, Carolina Oliveira de Souza, Elzânia Sales Pereira, Leilson Rocha Bezerra

**Affiliations:** 1 Department of Animal Science, Federal University of Campina Grande, Patos 58708110, Paraíba, Brazil; claudiney.felipe@estudante.ufcg.edu.br (C.F.A.I.); r.matheustavares98@gmail.com (R.M.T.d.O.); jmorais@cstr.ufcg.edu.br (J.M.P.F.); hkevily@gmail.com (K.H.d.O.S.d.L.); lucas-souza16@hotmail.com (L.d.S.B.); 2 Animal Science Department, Federal University of Bahia, Salvador 40170110, Bahia, Brazil; ronaldooloiveira@ufba.br (R.L.O.); claudioribeiro@ufba.br (C.V.D.M.R.); 3 Pharmacy Department, Federal University of Bahia, Salvador 40170155, Bahia, Brazil; carolods@ufba.br; 4 Animal Science Department, Federal University of Ceará, Fortaleza 60020181, Ceará, Brazil; elzania@hotmail.com

**Keywords:** biohydrogenation, lysine, *Longissimus* muscle, tannin, total phenol

## Abstract

This study investigated whether supplying rumen-protected lysine (RPL), alone or in combination with tannins, could modify the fatty acid (FA) profile, physicochemical characteristics, carcass traits, and sensory attributes of lamb meat. Forty Santa Inês × Dorper lambs (≈23 kg, 4 months old) were assigned to four dietary treatments for 55 days: a control diet, free lysine (0.44%), RPL microencapsulated in a carnauba-wax matrix, and RPL + tannins blend (1.34%). Feed intake, carcass weight, and quantitative carcass measurements did not differ among treatments (*p* > 0.05). Likewise, pH, color, proximate composition, water-holding capacity, cooking losses, and shear force remained unchanged. Dietary supplementation influenced the FA composition of the meat. RPL, especially when added with tannins, increased concentrations of conjugated linoleic acid (C18:2 *cis–9*, *trans–11*), eicosapentaenoic (C20:5 *n–3*), and docosahexaenoic acids (C22:6 *n–3*), improving the *n–6:n–3* ratio (*p* < 0.05). The sum and ratio of other FA and cardiometabolic indices were not altered. Lipid oxidation was reduced in RPL treatments, indicating enhanced oxidative stability. Sensory attributes scores were not affected (*p* > 0.05), ranging from “liked slightly” to “liked very much”. RPL, particularly when combined with tannins, improved specific health-related FA without adversely affecting carcass characteristics or consumer acceptance.

## 1. Introduction

Red meat significantly contributes to human diets by providing high-quality proteins and essential nutrients [1]. However, contemporary consumers increasingly prioritize products with improved nutritional attributes, particularly meat with healthier lipid profiles [2]. Concerns about the link between saturated fatty acid (SFA) intake and metabolic disorders have prompted exploration of dietary strategies to enhance unsaturated fatty acid (UFA) content in ruminant tissues while maintaining animal performance [3,4]. According to Lu et al. [5], meat quality is determined by structural and compositional characteristics that can be evaluated through physicochemical, microbiological, and sensory parameters.

In feedlot lamb production, precise nutritional management is crucial to ensure consistent growth and desirable carcass characteristics [6]. Essential amino acids, such as lysine, are key components of muscle development; however, their extensive degradation in the rumen limits their availability for absorption [7,8]. Technologies that protect amino acids from premature microbial breakdown have therefore become important tools for improving metabolizable protein supply and potentially influencing carcass and meat traits [9,10].

Condensed tannins are another nutritional resource with recognized capacity to alter rumen fermentation, reduce nitrogen losses, and modulate lipid metabolism through shifts in biohydrogenation pathways [11]. Their secondary antioxidant effects may also contribute to improved oxidative stability and visual quality of meat [12,13]. Although both protected amino acids and tannins have been documented to have benefits [9,10,14], the potential synergistic effect between these two approaches for improving lamb meat quality remains insufficiently characterized [15,16].

Thus, tannin addition has been associated with improvements in growth performance and nutrient use efficiency in small ruminants [17,18,19,20]. Moreover, tannins can alter ruminal biohydrogenation pathways, favoring the accumulation of beneficial FA such as polyunsaturated fatty acid (PUFA) and conjugated linoleic acid (CLA) in muscle tissues [21,22]. Their antioxidant properties further contribute to improved oxidative stability, color preservation, and extended shelf life of meat products [23,24].

Although the individual effects of rumen-protected amino acids and tannins have been investigated [9,10], there is limited information regarding their combined use, particularly in relation to lipid metabolism, oxidative stability, and physicochemical traits of lamb meat. The interaction between rumen-protected lysine (RPL) and natural tannins may offer complementary benefits for meat quality, yet this remains poorly documented in the available literature.

Given this context, the present study evaluated whether supplemental lysine, provided either in free form or microencapsulated in a carnauba wax matrix, and its combination with natural tannins can influence carcass traits and the physicochemical and lipid characteristics of lamb meat. This work aims to clarify the responses of muscle composition and FA profile to RPL, contributing to refined protein-nutrition strategies in intensive lamb production.

## 2. Materials and Methods

### 2.1. Ethical Considerations, Animals, Experimental Design, and Facilities

All experimental procedures were conducted in accordance with institutional animal welfare regulations and were approved by the Ethics Committee on Animal Use of the Federal University of Paraíba, Patos, Brazil (Protocol CEUA 35/2023). Forty non-castrated male lambs, Santa Inês × Dorper crossbreed, approximately four months old and averaging 23 ± 1.2 kg of body weight at the start of the trial, were distributed in a randomized block design with four dietary treatments, with ten lambs per treatment.

The experimental treatments consisted of four diets: control group received a basal ration without lysine supplementation; free lysine treatment included the same basal ration supplemented with 0.44% unprotected lysine; rumen-protected lysine (RPL) treatment received the basal ration containing 1.34% lysine microencapsulated in a carnauba-wax lipid matrix; finally, the RPL + tannins treatment offered the microencapsulated lysine at 1.34%, combined with tannins extracted from *Mimosa tenuiflora*, following the procedures recommended by Inô et al. [9].

Prior to commencing the feeding trial, lambs were identified, weighed, dewormed, and vaccinated against clostridial diseases and rabies. Animals were then housed individually in elevated wooden pens (1.3 × 1.5 m, suspended 0.5 m above the floor), each equipped with an individual feed trough and automatic drinker. Pens were arranged inside an open-sided barn (16 × 6 m) with slatted flooring and asbestos-cement roofing, ensuring adequate ventilation and protection from direct sunlight. The experiment lasted a total of 70 days, comprising a 15-day period for dietary and environmental adaptation followed by 55 days of data collection.

### 2.2. Microencapsulated Systems

The natural tannin used in the trial was extracted from *Mimosa tenuiflora* leaves and branches (≤8 mm diameter) collected at the vegetative growth stage, following the method described by Chaves [25]. For the RPL treatments, microencapsulation was performed using carnauba wax as the protective matrix. The coating process followed the fusion–emulsification technique outlined [9,10], producing stable lipid microcapsules suitable for ruminal protection.

### 2.3. Ingredients, Diets, and Chemical Composition

The diets were supplied as total mixed rations (TMRs) containing a 40:60 forage-to-concentrate ratio and formulated to support an average daily gain of 250 g/d in accordance with NRC [26] recommendations. The amounts of lysine added to the diets were determined based on metabolizable protein requirements described by the NRC [26] and BR-Meat Goats and Sheep [8]. All experimental diets were designed to be isoenergetic and isonitrogenous.

The forage component consisted of Tifton-85 (*Cynodon* spp.) and Buffel grass (*Cenchrus ciliaris* L.) hays. The concentrate portion was composed of ground corn, soybean meal, and a commercial mineral premix (Table 1). The TMR was offered twice daily at 08:00 and 15:00 h, allowing for approximately 10% feed refusals. Fresh water was available ad libitum throughout the trial.

Feed ingredients, refusals and experimental diets (Table 2) were dried, ground in a Wiley mill equipped with a 1 mm sieve, and analyzed using AOAC [27] procedures. Determinations included dry matter (DM; method 967.03), ash (method 942.05), crude protein (CP; Kjeldahl method), and ether extract (EE; method 920.29). Fiber fractions were assessed following Van Soest et al. [28], with procedural adaptations from Senger et al. [29]. Neutral detergent fiber (NDF) was determined using thermostable α-amylase and reported ash-free (aNDF). Lignin content was quantified using 72% sulfuric acid according to the AOAC method 973.18, as described by Licitra et al. [30]. Non-fibrous carbohydrates (NFC) were calculated using the equation proposed by Mertens [31]. Total digestible nutrients (TDN) were estimated from the relationship between TDN intake and DM intake [10]. Metabolizable energy (ME) was calculated as described by Weiss and Tebbe [32], assuming 4409 kcal of digestible energy (DE) per kg of TDN.

### 2.4. Performance, Slaughter Procedures, and Carcass Traits

At the conclusion of the 55-day feeding period, lambs were subjected to a 16 h fasting period, during which water remained available. Following the withdrawal period, slaughter body weight (SBW) was measured immediately prior to harvesting. Slaughter took place at a federally inspected facility (SIF) in accordance with established humane handling and animal welfare procedures [33]. Animals were rendered unconscious with a captive-bolt stunner, exsanguinated via severing of the jugular vein and carotid artery, and processed through hide removal, evisceration, and disarticulation of the head and lower limbs.

Hot carcass weight (HCW) was measured immediately after dressing. Carcasses were then chilled at 4 °C for 24 h, after which cold carcass weight (CCW) was obtained.

Carcass traits, hot carcass yield (HCY), cold carcass yield (CCY), and cooling loss (CCL) were calculated using Equations (1)–(3) according to Cartaxo et al. [34]:

(1) HCY = (HCW/SBW) × 100 

(2) CCY=(CCW/SBW)×100 

(3) CCL=(HCW−CCW)/HCW×100 

Carcasses were split along the vertebral column using an electric band saw (Ki Junta^®^, São Paulo, Brazil), and *Longissimus lumborum* samples were collected from the left half-carcass at the 12–13th rib interface.

Tissue samples were carefully trimmed to remove visible external fat, fascia, and connective tissues. Each sample was immediately packaged in airtight containers to minimize oxidative deterioration and microbial growth, properly labeled, and stored at −18 °C until further physicochemical analyses were conducted. Additional portions of the *Longissimus lumborum* muscle were freeze-dried, vacuum-sealed, and preserved under frozen conditions for subsequent fatty acid determination.

Muscle pH and temperature were assessed at two postmortem time points, immediately after slaughter (0 h) and after 24 h of refrigerated storage. Measurements were performed in triplicate using a digital potentiometer (Digimed 300M, Digimed, São Paulo, Brazil) equipped with a penetration probe inserted directly into the muscle tissue. Before each measurement session, the instrument was calibrated at 20 °C with standard buffer solutions of pH 4.0 and 7.0, following the manufacturer’s specifications to ensure analytical accuracy.

### 2.5. Physicochemical Characteristics and Proximate Composition

Color measurements were obtained immediately after pH evaluation on muscle portions carefully trimmed to remove any visible connective tissue. Steaks approximately 3.0 cm thick were cut perpendicular to muscle fiber direction and allowed to oxygenate at 4 °C for 30 min to promote uniform myoglobin blooming and stabilization of oxy-myo-globin pigments [35]. After this standardization period, surface color was recorded at three independent points using a portable chromameter (Konica Minolta Chroma Meter CR-410, Tokyo, Japan), which had been previously calibrated with a certified white tile in accordance with CIELAB guidelines (2° observer angle; 8 mm aperture). For each sample, mean values of lightness (L*), redness (a*), and yellowness (b*) were calculated. The chroma or saturation index (C*) was subsequently derived following Equation (4) [36]:

(4) $$C*\;={\left(a\;{*\;}^{2}\;+b\;{*\;}^{2}\right)}^{0.5}$$

The water retention capacity (WRC) was assessed using the Ham [37] methodology. Muscle cubes (5 g) were initially mass weighed (*W*1), positioned between filter paper sheets (Albert 238, 12.5 cm diameter) and dual acrylic plates, then subjected to compression under a 10 kg mass for 5 min. Post-compression, the samples and paper were reweighed (*W*2). *WRC* (%) was calculated from Equation (5):

(5) WRC = [(W2 − W1)/W2 × 100] 

Cooking weight loss (*CWL*) was determined following the guidelines of AMSA [38]. Two steak subsamples per animal (2.5 cm thickness, trimmed of all external fat) were weighed before cooking and then placed in an electric convection oven (Philco, Philadelphia, PA, USA) set at 170 ± 5 °C. Steaks were heated until they reached an internal temperature of 71 ± 5 °C, which was monitored using a stainless-steel thermocouple probe (Acurite) inserted into the geometric center of each sample. After cooking, steaks were allowed to equilibrate to room temperature (approximately 20 °C) before being weighed again. Cooking weight loss (%) was calculated as the proportion of mass lost during cooking relative to the initial raw weight, using Equation (6):

(6) CWL(%) = [ (Raw weight − Cooked weight)/ Raw weight ] × 100

Mechanical tenderness was assessed on the same cooked samples using the Warner–Bratzler shear force (WBSF) procedure. From each steak, three cylindrical cores (25 mm diameter) were removed with their longitudinal axis aligned with muscle fiber orientation. Each core was sheared perpendicularly to the fibers using a Warner–Bratzler blade attached to a texture analyzer (BFG 1000N, Mecmesin, UK). Before analysis, the device was calibrated with a certified 5 kg weight and operated at a crosshead speed of 20 cm/min. The average WBSF value from the three cores was used for statistical analysis, following the recommendations of AMSA [39].

Proximal composition was evaluated on *Longissimus lumborum* samples previously thawed for 20 h at 4 °C. Approximately 80 g of muscle tissue was homogenized using a multiprocessor (MICE-10, MICE, Sao Paulo, Brazil) to ensure uniform sample consistency. Moisture, crude protein, ash, lipid content, and collagen concentration were determined by near-infrared spectroscopy (NIR) using a FoodScan analyzer (FOSS Analytical A/S, Hillerød, Denmark). The instrument was operated under factory-calibrated prediction models, which were validated for use with meat products. Each sample was scanned in duplicate, and the internal software converted the spectral data into proximal composition outputs using established algorithms.

### 2.6. Lipid Oxidation

Lipid oxidation was quantified using the thiobarbituric acid reactive substances (TBARS) assay, following the analytical principles described by Botsoglou et al. [40]. Approximately 1.0 g of refrigerated Longissimus lumborum (stored at 4 °C for six days) was homogenized with 3 mL of deionized water in a tissue homogenizer (Turrax^®^, IKA-Werke, Staufenim Breisgau, Germany) operating at 9500 rpm for 45 s. Subsequently, 0.75 mL of trichloroacetic acid (25% *w*/*v*) was incorporated into the homogenate, and the mixture was vortexed for 15 min at 4 °C. The suspension was then centrifuged at 18,000× *g* for 15 min, also at 4 °C.

A 1.0 mL aliquot of the resulting supernatant was transferred to screw-cap borosilicate tubes and combined with 2.0 mL of thiobarbituric acid reagent (0.6% *w*/*v*). Samples were heated in a thermostatically controlled water bath at 70 °C for 30 min to allow chromogen formation. After cooling to room temperature, the absorbance of the solutions was measured at 532 nm using a UV–Vis spectrophotometer. Quantification of TBARS was based on a standard curve constructed from 1,1,3,3-tetraethoxypropane, and results were expressed as mg of malondialdehyde (MDA) equivalents per kg of muscle tissue [41].

### 2.7. Fatty Acid Meat Composition

Lipid extraction for fatty acid (FA) analysis in diets and *Longissimus lumborum* samples followed the method of Folch et al. [42]. Approximately 1 g of lyophilized muscle was homogenized in a mixture of methanol and chloroform using a Turrax homogenizer (15,000 rpm), and the extract was then filtered and washed with a potassium chloride solution. After phase separation and solvent removal under nitrogen at 37 °C, lipid residues were lyophilized and prepared for fatty acid methyl ester (FAME) derivatization according to O’Fallon et al. [43], using methanolic KOH and tridecanoic acid (C13:0) as internal standard.

Fatty acid methyl esters (FAMEs) were quantified using a gas chromatograph (Thermo Finnigan Trace-GC Ultra, Thermo Scientific, Waltham, MA, USA) fitted with a flame ionization detector and a highly polar CP-Sil 88 capillary column (100 m × 0.25 mm, 0.20 μm film thickness). Hydrogen served as the carrier gas at a flow rate of 1.8 mL/min. The oven temperature program was set from 70 °C to 230 °C, with a total analysis time of 65 min. Injector and detector temperatures were maintained at 250 °C and 300 °C, respectively. A 1.0 μL aliquot of sample extract was injected in each chromatographic run. Fatty acids were identified by comparison with certified retention time standards (Supelco™ 37 FAME Mix). Results are presented as g/100 g of total fatty acids.

From the chromatographic data, the following fatty acid groups and ratios were calculated: ΣSFA, ΣMUFA, ΣPUFA, *Σn–3*, *Σn–6*, and the MUFA: SFA, PUFA: SFA, and *n–6:n–3* ratios. Indices related to lipid nutritional quality, including the atherogenic index (AI), thrombogenic index (TI), the hypocholesterolemic: hypercholesterolemic ratio (h: H), and desirable fatty acids (DFA), were estimated according to methods described by Ulbricht and Southgate [44], Santos-Silva et al. [45], and Rhee [46]. Indices reflecting Δ9–desaturase activity (for C16 and C18 substrates) were calculated following the procedures outlined by Smet et al. [47].

### 2.8. Sensory Attributes

Steaks obtained from both sides of each carcass were portioned and frozen at −20 °C until they were subjected to sensory testing. From each hemicarcass, four steaks were collected (*n* = 8 per lamb), yielding a total of 320 samples from 10 animals. Sensory characteristics were assessed by a panel of 90 untrained consumers (45 men and 45 women, aged 18–54 years), generating 900 sensory observations [38].

Sensory evaluation followed the hedonic-scale protocol described by Amerine et al. [48], with each treatment assessed in triplicate. After thawing, samples (≈200 g) were cut into 1 cm^3^ cubes and cooked on an electric grill (George Foreman Jumbo Grill GBZ6BW, Rio de Janeiro, Brazil) preheated to 200 °C until they reached an internal temperature of 75 °C. Four cubes from each sample were transferred to pre-warmed, coded glass beakers, covered with watch glasses, and maintained in a water bath at 65–70 °C before serving.

Evaluations were conducted in individual sensory booths between 09:00 and 12:00 h. Ten panelists participated per session across nine sessions. Each panelist received eight samples (four treatments presented in duplicate), served in randomized order. Palate cleansers (filtered water and unsalted crackers) were provided. Participation procedures were approved by the Ethics Committee for Research Involving Human Subjects (Brazil Platform; CAAE: 71721623.6.0000.8035).

The sensory attributes evaluated included overall acceptability, tenderness, sheep-like aroma, flavor, sheep-like flavor, and juiciness [49,50]. A structured 9-point hedonic scale was used for all attributes, with anchors ranging from “dislike extremely” to “like extremely”, “extremely undesirable” to “extremely desirable”, “extremely tough” to “extremely tender”, “extremely dry” to “extremely juicy”, and “extremely intense” to “extremely weak”, depending on the attribute assessed.

### 2.9. Statistical Analysis

Data underwent statistical evaluation using a randomized complete block design incorporating four dietary interventions (control, free lysine, rumen-protected lysine (RPL), and RPL + tannin) with ten experimental replicates, analyzed through the MIXED procedure (SAS Institute Inc., version 9.4, Cary, NC, USA). The blocks were formed based on two ranges of initial weight of the lambs. Individual animals constituted the experimental unit for all response variables. The statistical framework is represented in Equation (7):

(7) Yij = μ + Bi + Dj + εij 

where *Yij* denotes the observed response, μ represents the grand mean, *Bi* corresponds to the block effect (i = 1–2), *Dj* indicates the dietary treatment effect (*j* = 14), and εij represents the random residual component. Initial body weight (BW) served as the blocking criterion for experimental unit allocation. Block effects were incorporated as random components in the statistical model. Treatment comparisons were conducted via Tukey’s test, establishing statistical significance at *p* ≤ 0.05.

Sensory attributes, including texture tenderness, moisture perception, flavor desirability, and overall acceptability, underwent analysis using linear mixed-effects modeling to identify determinants of consumer sensory response. Within this analytical framework, dietary treatments were designated as fixed effects, whereas individual evaluators and evaluation sessions were specified as random effects. Mean sensory scores for individual attributes were subjected to multiple comparison procedures using Tukey’s test, with a significance threshold established at *p* ≤ 0.05. For each dependent variable, optimal regression modeling was determined through minimization of the root mean square error (RMSE) criterion.

## 3. Results

Rumen-protected lysine (RPL) supplementation, whether in free form, microencapsulated, or co-encapsulated with tannin, exerted no significant effect (*p* > 0.05) on DM intake, slaughter body weight, hot and cold carcass weight and yield, cooling loss, or subcutaneous fat thickness (Table 3).

Most meat quality attributes (Table 4), including pH, color parameters, proximate composition, CWL, WBSF, and WRC, were not affected by dietary inclusion of RPL, either alone or combined with tannin (*p* > 0.05). Similarly, the proximate components of DM, moisture, ash, and crude protein did not differ among treatments (*p* > 0.05). However, the inclusion of RPL associated with tannin resulted in lower lipid oxidation in lamb meat compared with the control treatment (*p* < 0.01).

The fatty acid profile of the meat (Table 5) was also unchanged by the supplementation strategy (*p* > 0.05). The ƩSFA ranged from 44.75% in the free lysine treatment to 46.08% in the RPL–tannin blend, with palmitic acid (C16:0) and stearic acid (C18:0) remaining the predominant individual fatty acids across treatments.

The ƩUFA accounted for approximately 54–55% of the total FA, with oleic acid (C18:1 *cis–9*) representing the predominant component across all dietary treatments. Supplementation with RPL in combination with tannins resulted in significant increases (*p* < 0.05) in CLA (C18:2 *cis–9*, *trans–11*), EPA (C20:5 *n–3*), and DHA (C20:6 *n–3*) compared with the control group. This treatment also contributed to a reduction (*p* < 0.05) in the *n–6:n–3* ratio (Table 6).

Healthy lipidic indices and ratios were not affected by dietary treatments (*p* > 0.05). The ƩMUFA values ranged from 48.78% to 49.92%, whereas ƩPUFA levels varied between 5.14% and 5.39% without statistical changes. Although not statistically significant, the *n–6:n–3* ratio exhibited a numerical shift toward more desirable values with tannin inclusion (4.86 in the RPL–tannin group vs. 6.69 in the free-lysine treatment). Cardiometabolic indexes, including the AI, TI, and the h:H ratio, remained stable across treatments.

Estimated enzymatic activities, including Δ^9^-desaturase (C16 and C18 substrates), elongase, and Δ^9^-desaturase C16 activity h: H fatty acids ratio index in meat from lambs were also unaffected by treatment. Moreover, RPL supplementation, either alone or combined with condensed tannins, did not result in significant changes (*p* > 0.05) in consumer sensory evaluation of lamb meat attributes.

Overall acceptability (Table 7) ratings ranged from 6.79 in the RPL-tannin treatment to 7.55 in the control without statistical differentiation (*p* = 0.297).

Perceived tenderness scores remained consistently elevated across treatments, ranging between 7.58 (RPL and RPL-tannin) and 7.79 (control), showing no significant variation (*p* = 0.954). Similarly, species-specific aroma intensity was unaffected, varying between 6.68 (RPL-tannin) and 7.84 (free lysine; *p* = 0.157). Flavor desirability scores spanned from 6.89 (RPL-tannin) to 7.76 (control), demonstrating no treatment effect (*p* = 0.318). Species-specific flavor intensity also remained within a narrow range, from 6.53 (RPL) to 7.47 (control; *p* = 0.188). Moisture perception ratings varied between 6.84 (RPL) and 7.58 (control), with no significant inter-treatment differences (*p* = 0.411). All sensory attributes received ratings between 6.5 and 7.8 on the 9-point hedonic scale, corresponding to descriptive categories ranging from “slightly favorable” to “highly favorable”. No treatment generated mean scores below 6 (neutral threshold) or exceeding 8 (“extremely favorable”).

## 4. Discussion

The RPL supplementation, whether applied alone or in combination with condensed tannins, did not significantly affect slaughter body weight or carcass characteristics of lambs, including carcass weight and dressing percentage. This outcome suggests that lysine bioavailability was not a limiting factor for muscle accretion under the nutritional conditions of the current study. Indeed, when basal diets already meet or exceed metabolizable protein and essential amino acid requirements, additional RPL tends not to elicit measurable improvements in productive traits, as previously demonstrated by Frota et al. [51] and Araújo et al. [52]. The adequate protein supply in the experimental diets likely minimized the marginal benefit of further lysine supplementation. Moreover, the absence of a performance response [9,10] aligns with established metabolic principles, i.e., the anabolic effect of post-ruminal amino acid delivery depends on the synchronization between absorbed amino acids and available energy at the tissue level. When this synchrony is not limiting, extra lysine does not necessarily translate into additional lean tissue deposition [8,11]. Likewise, the presence of tannins, although capable of modulating ruminal nitrogen dynamics, did not impair nutrient availability to an extent that would influence growth or carcass outcomes. This reinforces that neither lysine nor tannin inclusion disrupted the balance between ruminal fermentation, metabolizable protein supply, and the physiological pathways governing carcass development [7].

The absence of treatment effects on performance parameters further indicates that the amino acid profile of the basal diet provided an adequate balance to support the observed growth responses, as the animals reached the expected weight gain according to established nutritional requirements. Comparatively, Bandeira et al. [53] reported favorable effects of *Mimosa tenuiflora* hay on certain carcass traits in sheep, including hot and cold carcass dressing percentages (44.7% and 43.2%, respectively), values closely aligned with those observed in the present study (44.8% and 43.6%). This similarity reinforces that the inclusion of tannins at the evaluated levels did not impair nutrient utilization or carcass deposition.

Regarding meat quality, no significant differences were detected for pH, color parameters, proximate composition, tenderness, or CWL. These findings confirm that RPL supplementation, whether alone or combined with tannins, did not disrupt muscle biochemical processes related to postmortem glycolysis, WRC, or proteolytic enzyme activity. Comparable stability in meat quality traits has been documented by Grassi et al. [54] and Nascimento et al. [55], even when protected amino acids were incorporated into ruminant diets. The preservation of these characteristics is critical from a commercial standpoint, as consumers consistently prioritize visual appearance, tenderness, and juiciness when making purchasing decisions. Nutritional interventions that enhance lipid quality without compromising sensory attributes are highly desirable.

Cooling loss did not differ among dietary treatments, indicating that neither RPL nor tannins altered postmortem moisture dynamics or fat coverage to an extent that would influence evaporative losses during cooling. Bandeira et al. [53] reported cooling losses between 3% and 4%, while Martins et al. [56] noted that values may range from 1% to 7%, depending on factors such as refrigeration chamber conditions, carcass surface area exposure, and adipose tissue deposition. In agreement with these observations, the present study recorded mean cooling loss values of less than 3%, suggesting that the carcasses maintained adequate fat coverage to minimize dehydration during cooling. This interpretation aligns with Carvalho et al. [57], who emphasize that lower cooling losses reflect better carcass fat distribution, which enhances thermal insulation and protects muscle integrity during the initial cooling phase.

The WRC values ranged from 55% to 70%, consistent with expectations for feedlot-finished lambs [58], and did not differ among treatments, indicating that RPL, alone or combined with tannins, did not affect muscle protein functionality or postmortem processes governing moisture retention. This stability aligns with the observed pH values, as a lower pH is known to reduce WRC by impairing the water’s ability to remain bound within muscle fibers [59]. Likewise, WBSF was unaffected by dietary treatments, ranging from 45.0 to 45.4 N, and remaining within the normal range of 35–55 N, which suggests that lysine and tannin supplementation did not alter proteolytic activity or oxidation pathways known to influence muscle toughness [60,61]. The color intensity of the meat also remained stable across treatments. However, several factors can affect this parameter; pH exerts a predominant effect by modulating the oxygenation state of myoglobin and, consequently, surface color [62]. The overall consistency, tenderness, and color indicate that neither RPL nor tannins compromised the technological quality of lamb meat under the evaluated conditions.

The yellowness index (b*) is often associated with intramuscular lipid content; however, neither free nor RPL supplementation influenced this parameter, and all values remained within acceptable limits for hair sheep meat. Cooking loss likewise did not differ among treatments, indicating that dietary interventions did not alter WRC during heating. As described by Makkar [6], cooking loss is highly dependent on carcass pH, with more acidic conditions promoting protein denaturation and reduced WRC, patterns not observed in the present study using RLP, consistent with the stable WRC values previously discussed.

Regarding the chemical composition, no treatment effects were detected, corroborating the findings of Costa [63], who also reported unchanged proximate composition in sheep fed acacia tannins. Similarly, Chikwanha et al. [64] observed no alterations in CP or DM when including up to 30% grape pomace tannins. However, the EE and ash values in the present investigation were higher. This difference may be attributable to the lower tannin concentrations used here, which likely avoided adverse effects on feed intake and nutrient digestibility, thereby supporting normal fat deposition and ash content. Collectively, these outcomes demonstrate that the inclusion of RPL or RPL–tannin did not compromise the physicochemical attributes or visual quality of lamb meat.

In contrast to the stability observed in other quality traits, lipid oxidation (TBARS) showed an apparent dietary effect, with the RPL–tannin treatment presenting significantly lower values (1.89 mg MDA/kg) compared with the control, lysine, and RPL groups (2.57, 2.58, and 2.58 mg MDA/kg, respectively). Although the TBARS values in the non-tannin treatments were slightly elevated, likely due to storage and refrigeration conditions during analysis [41], the reduction obtained with tannin supplementation indicates enhanced oxidative stability. This outcome aligns with the known antioxidant properties of tannins, which can chelate pro-oxidant metals and scavenge free radicals, thereby inhibiting lipid peroxidation [18,65,66]. While values above 2.5 mg MDA/kg may already signal the onset of perceptible oxidative changes [41,60], the decrease observed in the tannin group suggests a meaningful improvement in shelf life and sensory preservation. Overall, the combination of RPL–tannins demonstrated potential to reduce oxidative deterioration in lamb meat, reinforcing the role of dietary phenolics in promoting meat stability during storage [67].

The RPL alone or the RPL–tannin blend did not substantially alter the overall FA profile of lamb meat, as the proportions of SFA, MUFA, and PUFA remained within the expected ranges for the species [68]. However, the RPL–tannin treatment resulted in clear increases in CLA (C18:2 *cis–9*, *trans–11*), EPA (C20:5 *n–3*), and DHA (C20:6 *n–3*) compared with the control group. These improvements indicate that dietary tannins contributed to the retention of nutritionally important UFA, even though the broader lipid classes were unaffected [18,69]. Importantly, the *n–6:n–3* ratio was also reduced in the RPL–tannin group, reinforcing the enhancement in long-chain *n*–*3*PUFA deposition [70,71]. Lipid nutritional indices (AI, TI, and h:H ratio indexes) remained unchanged across treatments, demonstrating that these improvements occurred without disrupting the overall balance of health-related fatty acids [72,73].

Sensory evaluation revealed that the inclusion of RPL, either alone or in combination with tannins, did not impact consumer acceptance, as all attributes received scores ranging from “slightly favorable” to “highly favorable”. The stability in overall acceptability, tenderness, juiciness, aroma intensity, and flavor desirability demonstrated that these dietary interventions did not compromise palatability [74]. These findings align with previous studies indicating that rumen-protected amino acids generally exert minimal influence on organoleptic properties, as their primary effects relate to nutrient utilization rather than flavor precursor formation [54,55].

Although tannins can be associated with astringency, the levels used in this study did not negatively affect sensory perception, consistent with reports demonstrating that moderate tannin inclusion can improve oxidative stability without impairing consumer acceptance [67]. The slight numerical reduction observed in species-specific aroma and flavor intensity in the RPL–tannin treatment was not statistically significant and does not indicate any detrimental impact on sensory quality. Similar results were reported by Luciano et al. [67], who demonstrated that moderate inclusion of tannin-rich feedstuffs improved the oxidative stability of lamb meat without impairing consumer acceptance. The marginally lower scores observed for species-specific aroma and flavor intensity in the RPL-tannin treatment, although lacking statistical significance, may suggest a minor modulation of volatile compounds, consistent with the protective role of tannins against lipid oxidation [18].

Overall, the stable sensory profile observed across treatments supports the feasibility of utilizing RPL, with or without tannins, as a nutritional strategy to enhance metabolic efficiency and oxidative stability while maintaining consumer acceptance of lamb meat. Collectively, these findings demonstrate that although RPL supplementation did not directly influence growth performance, quantitative carcass characteristics, or sensory attributes, the association with tannins provided evidence of benefits regarding meat oxidative stability and some desirable fatty acids in lamb meat. This outcome indicates a potentially strategic role for the combined use of rumen-protected amino acids and phenolic compounds in ruminant nutrition, particularly when aligning productivity objectives with end-product quality enhancement.

Based on the results obtained, future research should explore longer feeding periods or different inclusion levels of RPL and tannins to determine whether the observed improvements in CLA, EPA, and DHA deposition can be further enhanced. Follow-up studies using rumen fermentation models or in vivo rumen fistulation could clarify how tannins modulate specific steps of biohydrogenation and unsaturated fatty acid escape. Additionally, evaluating antioxidant capacity and volatile compound profiles during extended storage would help elucidate the mechanisms underlying the improved oxidative stability observed in meat. Finally, integrating metabolomic or lipidomic approaches may provide deeper insight into how amino acid supply interacts with lipid metabolism and muscle biochemistry, supporting the development of more targeted nutritional strategies.

This study also presents important limitations that should be acknowledged. First, the amino acid requirements for small ruminants, particularly for individual essential amino acids such as lysine and methionine, remain poorly defined in the literature, which constrains the precision of diet formulation and the interpretation of supplementation outcomes. Second, although microencapsulation aims to protect amino acids from ruminal degradation, direct confirmation of intestinal release and absorption remains challenging. Techniques to measure true small-intestinal digestibility in vivo are difficult to apply in sheep and goats, limiting our ability to determine the exact proportion of rumen-protected amino acids that reached the duodenum intact and were effectively digested. Future studies employing isotopic tracers, mobile bag methods, or advanced digestibility markers may help overcome these constraints and improve understanding of amino acid utilization in small ruminants.

## 5. Conclusions

The inclusion of rumen-protected lysine either in free form or microencapsulated in carnauba wax, with or without *Mimosa tenuiflora* tannins, did not affect carcass traits, proximate composition, or sensory attributes of feedlot-finished Santa Inês lambs. Although the overall fatty acid composition and lipid indices remained largely unchanged, tannin supplementation increased CLA and EPA concentrations, improved the *n–6:n–3* ratio, and slightly enhanced Δ9–desaturase activity. Together, rumen-protected lysine and tannins contributed to a more favorable lipid profile and greater oxidative stability without compromising performance or technological meat quality. This nutritional strategy shows potential for producing lamb meat with improved functional and commercial value.

## Figures and Tables

**Table 1:** Chemical and fatty acid composition of ingredients used in the experimental diets.

Chemical Composition (g/kg DM)	Tifton-85 Hay	Ground Corn	Soybean Meal	Soybean Oil	Tannin Extract
Dry matter (g/kg as fed)	824	821	834	996	971
Crude ash	61.7	11.4	64.6	-	32.3
Crude protein	61.6	91.6	503	-	5.60
Ether extract	11.3	32.2	28.9	997	1.6
aNDF ^1^	687	167	148	-	-
Acid detergent fiber	357	22.4	51.7	-	-
Non-fiber carbohydrates	126	710	326	-	-
Acid detergent lignin	47.9	3.1	92.7	-	-
Fatty acid composition (% of total fatty acids)					
C8:0	0.056	0.006	0.012	-	-
C12:0	1.926	0.009	-	0.201	-
C14:0	0.701	0.070	0.119	-	-
C15:0	0.328	0.016	0.044	-	-
C16:0	28.82	15.92	18.98	14.35	-
C16:1 *cis*–9	0.033	0.114	0.066	-	-
C17:0	0.446	0.068	0.107	0.100	-
C17:1	ND	ND	0.034	-	-
C18:0	2.381	2.121	3.785	4.153	-
C18:1 *trans*	0.009	-	-	-	-
C18:1 *cis*–9	2.887	30.02	12.745	23.32	-
C18:1 *cis*–11	0.681	2.288	1.926	-	-
C18:1 *cis*–12	0.320	1.242	1.029	-	-
C18:1 *cis*–13	0.154	0.567	0.558	-	-
C18:2 *cis*–9 *cis*–12	15.33	44.84	54.78	52.78	-
C20:0	0.592	0.393	0.126	0.152	-
C18:3 *n–6*	0.003	0.010	-	-	-
C18:3 *n–3*	38.47	0.948	4.654	9.971	-
C20:1	3.001	0.215	0.268	0.200	-
C22:0	0.601	0.138	0.147	-	-
C20:3 *n–6*	0.333	-	0.043	-	-
C23:0	0.244	0.037	-	-	-
C24:0	0.603	0.144	0.083	0.180	-
∑SFA	36.69	18.9	23.40	19.03	-
∑MUFA	7.084	34.43	16.63	23.21	-
∑PUFA	54.14	45.85	59.48	55.98	-
Undefinied (others) ^2^	2.084	0.826	0.493	1.790	-

^1^ aNDF is neutral detergent fiber assayed with a heat-stable amylase and expressed exclusive for residual ash; ^2^ Sum of minor FA; saturated fatty acids (SFAs); monounsaturated fatty acids (MUFAs), polyunsaturated fatty acids (PUFAs).

**Table 2:** Ingredients proportion, chemical and fatty acid composition of the experimental diets.

Item	Control	Free Lysine	RPL ^1^	RPL ^1^ + Tannin
Proportion of ingredients (% DM)				
Tifton-85 hay	20.0	20.0	20.0	20.0
Buffel grass hay	20.0	20.0	20.0	20.0
Ground corn	41.2	41.7	40.8	40.8
Soybean meal	17.5	16.5	16.5	16.5
Free lysine	0.00	0.44	0.00	0.00
^1^ Rumen-protected lysine	0.00	0.00	1.34	0.00
^1^ Rumen-protected lysine + Tannin	0.00	0.00	0.00	1.34
Mineral mixture ^2^	1.33	1.33	1.34	1.34
Chemical composition (g/kg DM)				
Dry Matter	89.7	89.7	89.8	89.8
Ashes	6.75	6.70	6.70	6.70
Crude protein	13.3	13.3	13.2	13.2
Ether extract	2.42	2.42	2.38	2.38
aNDF ^3^	51.99	51.62	51.32	51.32
Acid detergent fiber	24.4	24.3	24.2	24.2
Non-fiber carbohydrates	38.3	38.4	37.6	37.6
Total digestible nutrients	71.3	70.9	70.0	70.0
Metabolizable energy	2.57	2.56	2.53	2.53
Fatty acid composition (% of total fatty acids)				
C8:0	0.003	0.012	0.003	0.003
C12:0	0.009	0.012	0.005	0.005
C14:0	0.09	0.099	0.092	0.094
C15:0	0.021	0.024	0.024	0.021
C16:0	13.2	13.0	14.4	13.6
C16:1 *cis*–9	0.09	0.06	0.08	0.063
C17:0	0.078	0.089	0.079	0.085
C17:1	0.03	0.004	0.026	0.029
C18:0	3.489	3.331	3.428	3.561
C18:1 *trans*	0.024	0.041	0.002	0.011
C18:1 *cis*–9	19.92	20.55	19.36	18.57
C18:1 *cis*–11	2.407	2.202	2.034	1.985
C18:1 *cis*–12	1.436	1.097	1.141	1.026
C18:1 *cis*–13	0.695	0.627	0.548	0.491
C18:2 *cis*–9 *cis*–12	51.95	51.83	51.88	53.47
C20:0	0.282	0.314	0.292	0.266
C18:3 *n–6*	0.002	0.001	0.003	0.001
C18:3 *n–3*	4.128	4.658	4.601	5.147
C20:1	0.279	0.324	0.521	0.398
C22:0	0.241	0.241	0.246	0.303
C20:3 *n–6*	0.024	0.07	0.066	0.001
C23:0	0.029	0.001	0.035	0.042
C24:0	0.089	0.111	0.099	0.094
∑SFA	17.57	17.27	18.76	18.034
∑MUFA	24.88	24.91	23.71	22.58
∑PUFA	56.10	56.56	56.55	58.62
Undefinied ^4^ (others)	1.447	1.262	0.982	0.764

^1^ Rumen-protected lysine (RPL) encapsulated in a carnauba wax lipid matrix; and rumen-protected lysine combined with tannins as an adjuvant. ^2^ Guaranteed levels (per kg of active elements): calcium, 145 g (max,); phosphorus, 97.8 g; sulfur, 38.0 g; copper, 1810 mg; cobalt, 66.0 mg; iron, 2846 mg; iodine, 89.5 mg; manganese, 1774 mg; selenium, 14.9 mg; zinc, 4298 mg; fluorine, 968.0 mg (maximum); ^3^ aNDF is neutral detergent fiber assayed with a heat stable amylase and expressed exclusive of residual ash; ^4^ Sum of minor FA; saturated fatty acids (SFA); monounsaturated fatty acids (MUFA), polyunsaturatedfatty acids (PUFA).

**Table 3:** Carcass traits of lambs (*n* = 40) fed diets containing a control (no lysine), free lysine (0.44%), rumen-protected lysine (RPL; 1.34%), or RPL combined with tannins.

Item	Control	Lysine	RPL ^1^	RPL ^1^ + Tannin	SEM ^2^	*p*-Value ^3^
Dry matter intake (kg/d)	1.19	1.14	1.21	1.18	0.06	0.878
Initial body weight (kg)	23.78	22.57	23.33	23.47	1.19	0.903
Slaughter body weight (kg)	36.02	34.43	36.18	35.12	1.30	0.767
Hot carcass weight (kg)	16.30	15.38	16.20	15.74	0.71	0.783
Cold carcass weight (kg)	15.88	15.01	15.71	15.36	0.70	0.826
Hot carcass yield (%)	45.24	44.53	44.67	44.75	0.68	0.890
Cold carcass yield (%)	44.07	43.44	43.33	43.64	0.69	0.886
Cooling loss (%)	2.60	2.50	2.99	2.48	0.25	0.485
Subcutaneous fat thickness (cm)	1.64	1.46	1.65	1.84	0.16	0.423

^1^ Rumen-protected lysine (RPL) encapsulated in a carnauba wax lipid matrix; and rumen-protected lysine combined with tannins as an adjuvant; ^2^ SEM = Standard error of the mean; ^3^ Means with different letters on the line indicate difference between treatments by Tukey’s test (*p* ≤ 0.05).

**Table 4:** Meat quality parameters of lambs (*n* = 40) fed diets containing a control (no lysine), free lysine (0.44%), rumen-protected lysine (RPL; 1.34%), or RPL combined with tannins.

Variables	Control	Lysine	RPL ^1^	RPL ^1^ + Tannin	SEM ^2^	*p*-Value ^3^
Initial pH	6.52	6.65	6.41	6.63	0.07	0.101
Final (24 h) pH	5.42	5.36	5.27	5.34	0.06	0.426
Initial temperature (°C)	35.63	34.53	36.01	33.77	0.62	0.065
Final (24 h) temperature (°C)	19.10	19.40	19.70	19.57	0.23	0.335
Color indexes						
Lightness (L*)	38.0	40.3	40.1	39.2	0.62	0.061
Redness (a*)	19.9	19.2	19.7	19.5	0.26	0.295
Yellowness (b*)	0.98	0.94	0.87	1.03	0.17	0.921
Chroma (C*)	19.9	19.2	19.8	19.5	0.27	0.297
Proximate composition (g/100 g meat)						
Moisture	74.5	74.1	73.3	73.6	0.62	0.256
Dry matter	26.1	25.9	26.7	26.4	0.26	0.788
Ash	1.20	1.19	1.26	1.14	0.08	0.792
Protein	21.9	20.9	22.2	22.1	0.84	0.727
Fat	3.00	3.81	3.24	3.16	0.12	0.120
Water retention capacity, %	71.7	69.6	71.7	73.7	1.19	0.589
Cooking weight loss, %	28.3	30.4	28.3	26.3	1.75	0.435
WBSF ^4^ (N)	45.1	45.4	45.0	45.1	0.43	0.400
Lipid oxidation (TBARS) ^5^	2.57a	2.58a	2.57a	1.89b	0.009	<0.01

^1^ Rumen-protected lysine (RPL) encapsulated in a carnauba wax lipid matrix; and rumen-protected lysine combined with tannins as an adjuvant; ^2^ SEM = Standard error of the mean; ^3^ Means with different letters on the line indicate difference between treatments by Tukey’s test (*p* ≤ 0.05); ^4^ Warner-Bratzler Shear Force (Newton force); ^5^ TBARS = Thiobarbituric acid reactive substances in mg MDA/kg of meat.

**Table 5:** Carcass traits of lambs (*n* = 40) fed diets containing: control (no lysine), free lysine (0.44%), rumen-protected lysine (RPL; 1.34%), or RPL combined with tannins.

Fatty Acids (g/100 g Total Fatty Acids)	Control	Lysine	RPL ^1^	RPL ^1^ + Tannin	SEM ^2^	*p*-Value ^3^
C4:0	0.032	0.027	0.023	0.023	0.006	0.620
C10:0	0.181	0.181	0.166	0.180	0.015	0.634
C12:0	0.134	0.102	0.084	0.104	0.020	0.316
C14:0	1.893	2.013	1.953	1.980	0.091	0.789
C15:0	0.278	0.258	0.239	0.225	0.015	0.073
C16:0	23.48	23.51	23.83	24.09	0.464	0.748
C17:0	0.821	0.832	0.794	0.793	0.042	0.869
C18:0	16.12	15.59	15.89	16.61	0.413	0.358
C19:0	2.281	2.234	2.385	2.013	0.210	0.696
C14:1 *cis*–9	0.043	0.063	0.144	0.059	0.038	0.297
C15:1 *cis*–9	0.450	0.431	0.409	0.401	0.031	0.704
C16:1 *cis*–9	1.678	1.716	2.031	1.689	0.101	0.060
C17:1 *cis*–9	0.548	0.626	0.586	0.539	0.048	0.580
C18:1 *cis*–9	46.85	47.08	46.03	46.09	0.385	0.141
C18:2 *n*–6	3.094	3.049	3.153	3.034	0.222	0.981
C18:3 *n*–6	0.216	0.209	0.193	0.223	0.020	0.730
C18:2 *c*9*t*11– (CLA) ^4^	0.144 ^c^	0.181 ^b^	0.170 ^b^	0.235 ^a^	0.012	0.023
C20:2 *n*–6	0.296	0.357	0.334	0.316	0.034	0.640
C20:4 *n*–6	1.384	1.502	1.469	1.225	0.115	0.338
C20:5 *n*–3 EPA ^4^	0.054 ^b^	0.032 ^c^	0.071 ^ab^	0.108 ^a^	0.010	0.033
C20:6 *n*–3 DHA ^4^	0.02 ^b^	0.02b	0.04 ^ab^	0.06 ^a^	0.005	0.001

^1^ Rumen-protected lysine (RPL) encapsulated in a carnauba wax lipid matrix; and rumen-protected lysine combined with tannins as an adjuvant; ^2^ SEM = Standard error of the mean; ^3^ Means with different letters on the line indicate difference between treatments by Tukey’s test (*p* ≤ 0.05). ^4^ CLA: conjugated linoleic acid [(C18:2 cis-9. trans-11; (rumenic acid + isomers)]; EPA: eicosapentaenoic acid; DHA: docosahexaenoic acid.

**Table 6:** Lipid nutritional indices calculated from saturated and unsaturated fatty acid proportions in lambs (*n* = 40) fed diets containing: control (no lysine), free Lysine (0.44% unprotected lysine), rumen-protected lysine (RPL, 1.34% microencapsulated lysine), or RPL combined with tannins extracted from *Mimosa tenuiflora*.

Fatty Acids ^4^	Control	Lysine	RPL ^1^	RPL ^1^ + Tannin	SEM ^2^	*p*-Value ^3^
Sums (total)						
ƩSFA	45.25	44.75	45.40	46.08	0.502	0.311
ƩMUFA	49.57	49.92	49.21	48.78	0.465	0.344
ƩPUFA	5.19	5.33	5.39	5.14	0.331	0.943
Ʃ*n*–3	0.29	0.26	0.30	0.39	0.043	0.510
Ʃ*n*–6	1.38	1.50	1.47	1.23	0.115	0.338
Ratios						
ƩUFA:ƩSFA	1.21	1.24	1.21	1.17	0.024	0.327
ƩMUFA:ƩSFA	1.10	1.12	1.09	1.06	0.021	0.301
ƩPUFA:ƩSFA	0.11	0.12	0.12	0.11	0.008	0.889
ƩPUFA:ƩMUFA	0.10	0.11	0.11	0.11	0.007	0.954
Ʃ*n*–6:Ʃ*n*–3	5.66 ^ab^	6.69 ^a^	6.58 ^ab^	4.86 ^b^	0.988	0.029
Indexes						
Desirable fatty acids	70.88	70.85	70.49	70.53	0.416	0.857
hypocholesterolemic (h)	48.50	48.83	47.77	47.65	0.365	0.078
Hypercholesterolemic (H)	25.51	25.63	25.86	26.18	0.515	0.794
h/H index	1.91	1.91	1.86	1.82	0.049	0.502
Thrombogenicity (TI)	1.57	1.55	1.59	1.63	0.039	0.499
Atherogenicity (AI)	0.61	0.61	0.62	0.64	0.018	0.639
Enzymatic activity						
Δ^9^–desaturase C16	6.65	6.82	7.85	6.55	0.366	0.070
Δ^9^–desaturase C18	74.41	75.14	74.35	73.52	0.576	0.271
Elongase	71.46	71.31	70.54	70.87	0.475	0.497

^1^ Rumen-protected lysine (RPL) encapsulated in a carnauba wax lipid matrix; and rumen-protected lysine combined with tannins as an adjuvant; ^2^ SEM = Standard error of the mean; ^3^ Means with different letters on the line indicate difference between treatments by Tukey’s test (*p* ≤ 0.05). ^4^ SFA, saturated fatty acids; MUFA, monounsaturated fatty acids; PUFA, polyunsaturated fatty acids; *n*–9, omega–9 PUFA; *n*–6, omega–6 PUFA; *n*–3, omega–3 PUFA; h/H, Hypocholesterolemic and hypercholesterolemic fatty acids ratio; Atherogenicity index (IA); Thrombogenicity index (TI) and desirable fatty acids (DFA).

**Table 7:** Sensory attributes of lamb meat (*n* = 40) from animals fed Control, Free Lysine, rumen-protected lysine (RPL), or RPL + Tannins diets.

Variables ^1^	Control	Lysine	RPL ^2^	RPL ^2^ + Tannin	SEM ^3^	*p*-Value ^4^
Overall acceptance	7.55	7.50	7.11	6.79	0.321	0.297
Tenderness	7.79	7.71	7.58	7.58	0.314	0.954
Sheep-like aroma	7.34	7.84	7.24	6.68	0.355	0.157
Flavor	7.76	7.5	7.11	6.89	0.357	0.318
Sheep-like flavor	7.47	7.26	6.53	6.58	0.374	0.188
Juiciness	7.58	6.89	6.84	7.11	0.341	0.411

^1^ Hedonic scale (1 to 9) applied with 90 panelists according to Larmond [49] and Madruga [50]. ^2^ Rumen-protected lysine (RPL) encapsulated in a carnauba wax lipid matrix; and rumen-protected lysine combined with tannins as an adjuvant; ^3^ SEM = Standard error of the mean; ^4^ Means with different letters on the line indicate difference between treatments by Tukey’s test (*p* ≤ 0.05).

## Data Availability

The data that support the findings of this study are available from the corresponding author upon reasonable request.

## Abbreviations

The following abbreviations are used in this manuscript:

*a**	redness
ADF	acid detergent fiber
AI	atherogenic index
*b**	yellowness
CLA	conjugated linoleic acid
CP	crude protein
CWL	cooking weight loss
DHA	docosahexaenoic acid
DFA	desirable fatty acids
DM	dry matter
EE	ether extract
EPA	eicosapentaenoic acid
FAME	fatty acid methyl esters
FA	fatty acid
h:H	hypocholesterolemic and hypercholesterolemic fatty acids ratio
*L**	lightness
MCFA	medium-chain-FA
MUFA	summed monounsaturated fatty acids
n–3	omega-3
n–6	omega-6
n–9	omega-9
NFC	nonfibrous carbohydrate
NDF	neutral detergent fiber
aNDF	Neutral detergent fiber assayed with a heat-stable amylase and expressed exclusive for residual ash
PUFA	polyunsaturated fatty acids
SFA	saturated fatty acids
TI	thrombogenicity index
TDN	total digestible nutrients
UFA	total unsaturated fatty acids
WHC	water holding capacity

## References

[1] Zanovec M., O’neil C.E., Keast D.R., Fulgoni V.L., Nicklas T.A. (2010). Lean beef contributes significant amounts of key nutrients to the diets of US adults: National Health and Nutrition Examination Survey 1999–2004. Nutr. Res..

[2] Firetti R., Alberti A.L.L., Zundt M., Carvalho Filho A.A., Oliveira E.C. (2017). Identificação de Demanda e Preferências no Consumo de Carne Ovina com Apoio de Técnicas de Estatística Multivariada. Rev. Econ. Sociol. Rural.

[3] Osório J.C.S., Sañudo Astiz C., Osório M.T.M., Alfranca I.S. (1998). Produção de Carne Ovina, Alternativa para o Rio Grande do Sul.

[4] Kontogianni M.D., Panagiotakos D.B., Pitsavos C., Chrysohoou C., Stefanadis C. (2008). Relationship between meat intake and the development of acute coronary syndromes: The CARDIO2000 case–control study. Eur. J. Clin. Nutr..

[5] Correia B.R., Carvalho G.G.P., Oliveira R.L., PIRES A.J.V., Ribeiro O.L., Silva R.R., Carvalho B.M.A. (2016). Production and quality of beef from young bulls fed diets supplemented with peanut cake. Meat Sci..

[6] Makkar H.P.S. (2003). Effects and fate of tannins in ruminant *Anim*, adaptation to tannins, and strategies to overcome detrimental effects of feeding tannin-rich feeds. Small Rumin. Res..

[7] Patra A.K., Saxena J. (2011). Exploitation of dietary tannins to improve rumen metabolism and ruminant nutrition. J. Sci. Food Agric..

[8] BR-Meat, Goats and Sheep (2024). Nutritional Requirements of Goats and Sheep.

[9] Inô C.F.A., Pereira Filho J.M., de Oliveira R.M.T., de Oliveira J.F.P., da Silva Filho E.C., Nascimento A.M.d.S.S., Oliveira R.L., do Nascimento R.R., de Lucena K.H.d.O.S., Bezerra L.R. (2024). New Technology of Rumen-Protected Bypass Lysine Encapsulated in Lipid Matrix of Beeswax and Carnauba Wax and Natural Tannin Blended for Ruminant Diets. Animals.

[10] Oliveira R.M., Pereira Filho J.M., Inô C., Andrade É., Lucena K.H., Oliveira J.P., Pereira E., Oliveira R., Edvan R., Bezerra L. (2025). Microencapsulated Escape Lysine with Tannin as an Adjuvant in Sheep Diets. Vet. Sci..

[11] Sun Z.H., Tan Z.L., Liu S.M., Tayo G.O., Lin B., Teng B., Tang S.X., Wang W.J., Liao Y.P., Pan Y.F. (2007). Effects of dietary methionine and lysine sources on nutrient digestion, nitrogen utilization, and duodenal amino acid flow in growing goats. J. Anim. Sci..

[12] Fonseca N.V.B., Cardoso A.d.S., Bahia A.S.R.d.S., Messana J.D., Vicente E.F., Reis R.A. (2023). Additive Tannins in Ruminant Nutrition: An Alternative to Achieve Sustainability in Animal Production. Sustainability.

[13] Orlandi T., Kozloski G.V., Alves T.P., Mesquita F.R., Ávila S.C. (2015). Digestibility, ruminal fermentation and duodenal flux of amino acids in steers fed grass forage plus concentrate containing increasing levels of *Acacia mearnsii* tannin extract. Anim. Feed. Sci. Technol..

[14] Galvão J.M., Silva T.M., Silva W.P., Pimentel P.R.S., Barbosa A.M., Oliveira R.L. (2020). Intake, digestibility, ingestive behavior, and nitrogen balance of goats fed with diets containing residue from tamarindo fruit. Trop. Anim. Health Prod..

[15] Rira M., Morgavi D.P., Archimède H. (2015). Potential of tannin-rich plants for modulating ruminal microbes and ruminal fermentation in sheep. J. Anim. Sci..

[16] Frutos P., Hervás G., Natalello A., Luciano G., Fondevilla M., Priolo A. (2020). Ability of tannins to modulate ruminal lipid metabolism and milk and meat fatty acid profiles. Anim. Feed. Sci. Technol..

[17] Frutos P., Hervás G., Ramos G., Giráldez F.J., Mantecón A.R. (2002). Condensed tannin content of several shrub species from a mountain area in northern Spain, and its relationship to various indicators of nutritive value. Anim. Feed. Sci. Technol..

[18] Vasta V., Luciano G. (2011). The effects of dietary consumption of plants secondary compounds on small ruminants’ products quality. Small Rumin. Res..

[19] Mcallister T.A., Wang Y., Diarra M.S., Alexander T., Stanford K. (2018). Challenges of a one-health approach to the development of alternatives to antibiotics. Anim. Front..

[20] Lima P.R., Apdini T., Freire A.S., Santana A.S., Moura L.M.L., Nascimento J.C.S., Menezes D.R. (2019). Dietary supplementation with tannin and soybean oil on intake, digestibility, feeding behavior, ruminal protozoa and methane emission in sheep. Anim. Feed. Sci. Technol..

[21] Vasta V., Mele M., Serra A., Scerra M., Luciano G., Lanza M., Priolo A. (2009). Metabolic fate of fatty acids involved in ruminal biohydrogenation in sheep fed concentrate or herbage with or without tannins. J. Anim. Sci..

[22] Kamel H.E.M., Al-Dobaib S.N., Salem A.Z., López S., Alaba P.A. (2018). Influence of dietary supplementation with sunflower oil and quebracho tannins on growth performance and meat fatty acid profile of Awassi lambs. Anim. Feed. Sci. Technol..

[23] Grainger C., Clarke T., Auldist M.J., Beauchemin K.A., Mcginn S.M., Waghorn G.C., Eckard R.J. (2009). Potential use of *Acacia mearnsii* condensed tannins to reduce methane emissions and nitrogen excretion from grazing dairy cows. Can. J. Anim. Sci..

[24] Bellés M., Campo M.M., Roncalés P., Beltrán J.A. (2019). Supranutritional doses of vitamin E to improve lamb meat quality. Meat Sci..

[25] Chaves I.L.S. (2018). Extração e Caracterização de Taninos de Casca de Eucalipto Cultivada em Ambientes Contrastantes. Dissertação de Mestrado.

[26] National Research Council-NRC (2007). Nutrient Requirements of Small Ruminants: Sheep, Goats, Cervids, and New World Camelids.

[27] AOAC (2012). Official Methods of Analysis.

[28] Van Soest P.J., Robertson J.B., Lewis B.A. (1991). Métodos para fibra alimentar, fibra em detergente neutro e polissacarídeos não amiláceos em relação à nutrição animal. J. Dairy Sci..

[29] Senger C.C.D., Kozloski G.V., Bonnecarrère Sanchez L.M., Mesquita F.R., Alves T.P., Castagnino D.S. (2008). Evaluation of autoclave procedures for fibre analysis in forage and concentrate feedstuffs. Anim. Feed Sci. Technol..

[30] Licitra G., Hernandez T.M., Van Soest P.J. (1996). Standardization of procedures for nitrogen fractionation of ruminant feeds. Anim. Feed. Sci. Technol..

[31] Mertens D.R. (2002). Gravimetric determination of amylase-treated neutral detergent fiber in feeds with refluxing in beakers or crucibles: Collaborative study. J. AOAC Int..

[32] Weiss W.P., Tebbe A.W. (2019). Estimating digestible energy values of feeds and diets and integrating those values into net energy systems. Trans. Anim. Sci..

[33] Ministério de Agricultura Pecuária e Abastecimento (2000). Normativa n°03/00, de 07 de Janeiro de 2000. Aprova o Regulamento Técnico de Métodos de Insensibilização para o Abate Humanitário de Animais de Açougue.

[34] Cartaxo F.Q., Cezar M.F., Sousa W.H., Gonzaga Neto S.G., Pereira Filho J.M.P., Cunha M.G.G. (2009). Quantitative traits of carcass from lambs finished in feedlot system and slaughtered at different body conditions. Rev. Bras. Zootec..

[35] Mancini R., Kerth C.R. (2013). Meat Color. The Science of Meat Quality.

[36] Boccard R., Buchter L., Casteels E., Cosentino E., Dransfield E., Hood D.E., Joseph R.L., Macdougall D.B., Rhodes D.N., Schön I. (1981). Procedures for measuring meat quality characteristics in beef production experiments. Report of a working group in the Commission of the European Communities’(CEC) beef production research programme. Livest. Prod. Sci..

[37] Hamm R., Bechtel P.J. (1986). Functional properties of the myofibrillar-system and their measurements. Muscle as Food.

[38] AMSA (2015). Research Guidelines for Cookery, Sensory Evaluation, and Instrumental Tenderness Measurements of Meat.

[39] Shackelford S.D., Wheeler T.L., Koohmaraie M. (1999). Evaluation of slice shear force as an objective method of assessing beef longissimus tenderness. J. Anim. Sci..

[40] Botsoglou N.A., Fletorius D.J., Papageorgiu G.E., Vassilopoulos V.N., Mantis A.G., Trakatellis A.G. (1994). Rapid, sensitive, and specific thiobarbituric acid method for measuring lipid peroxidation in animal tissue, food, and feedstuff samples. J. Agric. Food Chem..

[41] Buege J.A., Aust S.D. (1978). Microsomal lipid peroxidation. Methods in Enzymology.

[42] Folch J., Lees M., Sloane Stanley G.H. (1957). A simple method for the isolation and purification of total lipides from animal tissues. J. Biol. Chem..

[43] O’Fallon J.V., Busboom J.R., Nelson M.L., Gaskins C.T. (2007). A direct method for fatty acid methyl ester (FAME) synthesis: Application to wet meat tissues, oils and feedstuffs. J. Anim. Sci..

[44] Ulbricht T.L., Southgate D.A. (1991). Coronary heart disease: Seven dietary factors. Lancet.

[45] Santos-Silva J., Mendes I.A., Bessa R.J.B. (2002). The effect of genotype, feeding system and slaughter weight on the quality of light lambs. 1. Growth, carcass composition and meat quality. Livest. Prod. Sci..

[46] Rhee K.S., Chow C.K. (1992). Fatty acids in meats and meat products. Fatty Acids in Foods and Their Health Implications.

[47] Smet S., Raes K., Demeyer D. (2004). Meat fatty acid composition as affected by fatness and genetic factors: A review. Anim. Res..

[48] Amarine M.A., Pangborn M.R., Roessler E.B. (1965). Principles of Sensory Evaluation off New York.

[49] Larmond E. (1977). Laboratory Methods for Evaluation of Foods.

[50] Madruga M.S. (1997). Revisão: Formação do aroma cárneo. Bol. Soc. Bras. Ciênc. Tecnol. Aliment..

[51] Frota H.N., Reis R.B., Faria B.N., Coelho S.G., Saturnino H.M. (2014). Supplementation of lysine and methionine in association or not with soybean oil in dairy cow diets. Arq. Bras. Med. Vet. Zootec..

[52] Araújo C.M., Macedo Júnior G.L., Oliveira K.A., Silva A.L., Santos M.T.S. (2020). Nutritional and metabolic evaluation of feeding lambs with growing levels Of protein amino acids. Arq. Bras. Med. Vet. Zootec..

[53] Bandeira P.A.V., Filho J.M.P., de Azevêdo Silva A.M., Cezar M.F., Bakke O.A., Silva U.L., Bezerra L.R. (2017). Performance and carcass characteristics of lambs fed diets with increasing levels of Mimosa tenuiflora (Willd.) hay replacing Buffel grass hay. Trop. Anim. Health Prod..

[54] Grassi G., Di Gregorio P., Capasso G., Rando A., Perna A.M. (2024). Effect of dietary supplementation with rumen-protected amino acids, lysine and methionine, on the performance of Comisana ewes and on the growth of their lambs. Anim. Sci. J..

[55] Nascimento T.V.C., Macedo Junior G.L., Silva D.C., Oliveira L.S. (2017). Rumen-protected amino acids in ruminant nutrition: A review. Rev. Bras. Zootec..

[56] Jones A.G., Takahashi T., Fleming H., Griffith B.A., Harris P., Lee M.R.F. (2021). Using a lamb’s early-life liveweight as a predictor of carcass quality. Animals.

[57] Carvalho I.P., Fiorentini G., Lage J.F., Messana J.D., Canesin R.C., Rossi L.G., Berchielli T.T. (2017). Fatty acid profile, carcass traits and meat quality of Nellore steers following supplementation with various lipid sources. Anim. Prod. Sci..

[58] Hopkins D.L., Hegarty R.S., Walker P.J., Pethick D.W. (2006). Relationship between animal age, intramuscular fat, cooking loss, pH, shear force and eating quality of aged meat from sheep. Aust. J. Exp. Agric..

[59] Gomide L.A.M., Ramos E.M., Fontes P.R. (2013). Ciência e Qualidade da Carne: Fundamentos.

[60] Lund M.N., Heinonen M., Baron C.P., Estévez M. (2011). Protein oxidation in muscle foods: A review. Mol. Nutr. Food Res..

[61] Brewer S. (2010). Technological quality of meat for processing. Handbook of Meat Processing.

[62] Cézar M.F., Sousa W.H. (2007). Carcaças ovinas e caprinas obtenção, avaliação e classificação. Ed. Agropecu. Trop..

[63] Costa E.I.S. (2019). Condensed Tannins in the Diet of Lambs. Ph.D. Thesis.

[64] Chikwanha O.C., Muchenje V., Nolte J.E., Dugan M.E.R., Mapiye C. (2019). Grape pomace (*Vitis vinifera* L. cv. *Pinotage*) supplementation in lamb diets: Effects on growth performance, carcass and meat quality. Meat Sci..

[65] Lee J.H., Min B.R., Lemma B.B. (2017). Quality characteristics of goat meat as influenced by condensed tannins-containing pine bark. Small Rumin. Res..

[66] Pereira Filho J.M., Vieira E.L., Kamalak A., Silva A.M.A., Cézar M.F., Beelen P.M.G. (2005). Correlação entre o teor de tanino e a degradabilidade ruminal da matéria seca e proteína bruta do feno de jurema-preta (Mimosa tenuiflora Wild) tratada com hidróxido de sódio. Livest. Res. Rural Dev..

[67] Luciano G., Monahan F.J., Vasta V., Biondi L., Lanza M., Bella M., Pennisi P., Priolo A. (2009). Lamb meat colour stability as affected by dietary tannins. Ital. J. Anim. Sci..

[68] Jenkins T.C., Wallace R.J., Moate P.J., Mosley E.E. (2008). Board-invited review: Recent advances in biohydrogenation of unsaturated fatty acids within the rumen microbial ecosystem. J. Anim. Sci..

[69] Buccioni A., Decandia M., Minieri S., Molle G., Cabiddu A. (2015). Lipid metabolism in the rumen: New insights on lipolysis and biohydrogenation with an emphasis on the role of endogenous plant factors. Anim. Feed. Sci. Technol..

[70] Scollan N., Hocquette J.F., Nuernberg K., Dannenberger D., Richardson I., Moloney A. (2006). Innovations in beef production systems that enhance the nutritional and health value of beef lipids and their relationship with meat quality. Meat Sci..

[71] Miltko R., Majewska M.P., Bełżecki G., Kula K., Kowalik B. (2019). Growth performance, carcass and meat quality of lambs supplemented with different vegetable oils. Asian-Australas. J. Anim. Sci..

[72] Palmquist D.L., Lock A.L., Shingfield K.J., Bauman D.E. (2005). Biosynthesis of conjugated linoleic acid in ruminants and humans. Adv. Food Nutr. Res..

[73] Shingfield K.J., Bonnet M., Scollan N.D. (2013). Recent developments in altering the fatty acid composition of ruminant-derived foods. Animals.

[74] Wood J.D., Enser M., Fisher A.V., Nute G.R., Sheard P.R., Richardson R.I., Hughes S.I., Whittington F.M. (2008). Fat deposition, fatty acid composition and meat quality: A review. Meat Sci..

